# Malignant bone tumors (other than Ewing’s): clinical practice guidelines for diagnosis, treatment and follow-up by Spanish Group for Research on Sarcomas (GEIS)

**DOI:** 10.1007/s00280-017-3436-0

**Published:** 2017-10-16

**Authors:** Andrés Redondo, Silvia Bagué, Daniel Bernabeu, Eduardo Ortiz-Cruz, Claudia Valverde, Rosa Alvarez, Javier Martinez-Trufero, Jose A. Lopez-Martin, Raquel Correa, Josefina Cruz, Antonio Lopez-Pousa, Aurelio Santos, Xavier García del Muro, Javier Martin-Broto

**Affiliations:** 1 Hospital La Paz, Paseo Castellama, 261, 28046 Madrid, Spain; 2 Hospital de la Santa Creu i Sant Pau, Carrer de Sant Quintí, 89, 08026 Barcelona, Spain; 3 Hospital Vall d’Hebrón, Passeig de la Vall d’Hebrón 119-129, 08035 Barcelona, Spain; 4 Hospital Gregorio Marañón, C/ Dr Esquerdo 46, 28007 Marid, Spain; 5 Hospital Miguel Servet, Paseo Isabel la Católica 1-3, 50009 Zaragoza, Spain; 6 Hospital 12 de Octubre, Ctra, Andalucía, km. 5.4, 28041 Madrid, Spain; 7 Hospital Virgen de la Victoria, Campus de Teatinos s/nº, 29010 Málaga, Spain; 8 Hospital Universitario de Canarias, Carretera de Ofra s/n, 38320 San Cristóbal de la Laguna, Santa Cruz De Tenerife Spain; 9 Hospital de la Santa Creu i Sant Pau, C/. Mas Casanovas, 90, 08041 Barcelona, Spain; 10 Hospital Virgen del Rocío, Av Manuel Siurot s/n, 41013 Sevilla, Spain; 11 Institut Catalá d’Oncologia Hospitalet, Avinguda de la GranVia de l´Hospitalet 199-203, L´Hospitalet de Llobregat, Barcelona, Spain

**Keywords:** Bone tumors, Bone sarcomas, Clinical guideline, Diagnosis, Treatment

## Abstract

Primary malignant bone tumors are uncommon and heterogeneous malignancies. This document is a guideline developed by the Spanish Group for Research on Sarcoma with the participation of different specialists involved in the diagnosis and treatment of bone sarcomas. The aim is to provide practical recommendations with the intention of helping in the clinical decision-making process. The diagnosis and treatment of bone tumors requires a multidisciplinary approach, involving as a minimum pathologists, radiologists, surgeons, and radiation and medical oncologists. Early referral to a specialist center could improve patients’ survival. The multidisciplinary management of osteosarcoma, chondrosarcoma, chordoma, giant cell tumor of bone and other rare bone tumors is reviewed in this guideline. Ewing’s sarcoma will be the focus of a separate guideline because of its specific biological, clinical and therapeutic features. Each statement has been accompanied by the level of evidence and grade of recommendation on the basis of the available data. Surgical excision is the mainstay of treatment of a localized bone tumor, with various techniques available depending on the histologic type, grade and location of the tumor. Chemotherapy plays an important role in some chemosensitive subtypes (such as high-grade osteosarcoma). In other subtypes, historically considered chemoresistant (such as chordoma or giant cell tumor of bone), new targeted therapies have emerged recently, with a very significant efficacy in the case of denosumab. Radiation therapy is usually necessary in the treatment of chordoma and sometimes of other bone tumors.

## Introduction

Primary bone tumors are an uncommon and heterogeneous group of malignancies. Osteosarcoma is the most frequent with an estimated incidence average of 0.2–0.3 cases per 100,000 per year in Europe. It occurs mainly in adolescents and young adults, but is also seen in older patients, usually related to prior radiotherapy or Paget’s disease. Chondrosarcoma is the most frequent bone sarcoma in adults, with an incidence average of 0.2 cases per 100,000 per year. It often has an aggressive local behavior but rarely metastasizing, although in some cases more aggressive forms can also be seen. Chordoma is a very uncommon primary tumor of bone with invasive local behavior and metastatic potential, mostly after multiple local recurrences. Giant cell tumor of bone (GCTB) is a rare tumor, affecting mainly young adults. Although often considered benign, in some cases it can metastasize and/or suffer malignant transformation. Lastly, Ewing’s sarcoma is a tumor of children and adolescents that because of its specific biological, clinical and therapeutic features will be the focus of a different guideline.

Due to the complexity and rarity of bone tumors, Spanish Group for Research on Sarcoma (GEIS) decided to develop this clinical guideline with the aim of providing practical recommendations to help in the clinical decision-making process and improve outcomes for bone sarcoma patients.

Diagnosis and treatment of bone tumors requires a multidisciplinary approach, involving as a minimum pathologists, radiologists, surgeons, and radiation and medical oncologists. There is a great deal of evidence demonstrating that early recognition and referral to a specialist center that provides a multidisciplinary diagnosis and therapeutic approach, and manages a high number of cases annually, could improve outcomes in patients with bone tumors. Therefore, centralized referral should ideally be done from the moment a diagnosis of a bone sarcoma is suspected (III, A) [1].

## Methods

These guidelines have been developed by a multidisciplinary panel of specialists in the different fields involved in the diagnosis and treatment of bone tumors. A bibliographic search of published articles was performed in the PubMed database. In a face-to-face consensus meeting, each section was presented by one expert to the entire group, for a discussion and consensus of the statements. The panel adopted the American Society of Clinical Oncology (ASCO) levels of evidence/grades of recommendation [2].

## Diagnosis and staging

### Imaging

The initial approach to any suspected bone lesion must be with radiographic plain films taken in two orthogonal planes. In spite of more sophisticated imaging technologies such as Magnetic Resonance Imaging (MRI), plain film continues to offer canonical elements in the diagnostic process of bone tumors [3].

MRI is the next imaging step which allows both, a narrower differential diagnosis and local staging. The imaging protocol should include sequences T1-Spin-Echo (SE) and T2-Fast-SE (FSE) weighted images (WI) with chemical fat suppression (FS) in the axial plane and in sagittal or coronal planes. Short-TI Inversion Recovery (STIR) sequences also allow good T2 contrast, and they are recommended when susceptibility artifacts are present, in at least one plane.

MRI is also the main tool for local staging of primary bone tumors and some important rules should be borne in mind when it is performed, before any treatment: (a) it must include, at some point in the process, the two joints either side of the tumor in the coronal plane; (b) careful attention should be paid to any epiphyseal or articular invasion, especially in young patients without physeal closure; (c) STIR sequences should not be used to measure tumor extension, since they can lead to overestimation in both intramedullary and soft tissue (IV, A); (d) it is desirable that any follow-up with MRI uses the same protocol every time, as it will enable a better comparison between them (IV, B).

Use of gadolinium contrast enhancement, static (CE-MRI) or dynamic (DCE-MRI), in diagnosis and evaluation of bone tumor is controversial, but it has two potential advantages: (a) it allows adequate planning of biopsy route avoiding necrotic, hemorrhagic or low-grade sarcoma areas, and (b) it allows monitoring of the response to chemotherapy (IV, B). CE-MRI study requires an axial or coronal pre-contrast T1-SE WI with fat suppression (FS) sequence and the same after contrast. New MR diffusion-weighted images (MR-DWI) have become increasingly used in the evaluation, staging and follow-up of bone sarcomas [4].

Neither MR-DWI nor DCE-MRI have demonstrated any benefit for diagnostic purposes but there is some evidence of promising use when applied to response evaluation of neoadjuvant therapies (mainly DCE-MRI) and for tumor staging and follow-up, as well as for searching for local recurrences or metastases other than lungs (mainly MR-DWI) (IV, B). To make the most of these procedures, they should be performed at the time of basal protocol.

Computed tomography (CT) of the primary bone tumor is not always necessary for diagnosis, but CT can help in some instances to evaluate bone matrix or calcifications, especially when chondroid tumors are suspected, or in chordoma (IV, A). Additionally, CT can be of value for planning surgery in complex areas like pelvis or spine (V, A). The key imaging diagnostic elements to analyze at baseline are summarized in Table 1.

**Table 1:** Diagnostic decision-making checklist for bone tumors

Criteria	Elements
Demographics	Age Gender
Affected bone	Single or multiple Long, short or flat
Localization in longitudinal axis	Epiphyseal Metaphyseal Diaphyseal
Localization in axial axis	Central Eccentrical Cortical Yuxtacortical
Destruction pattern	Geographical (lytic, sclerotic or mixed) Infiltrative (lytic, sclerotic or mixed) Moth-eaten
Periosteal reaction	Sunburst Hair-on-end Codman’s triangle Onion-skin
Bone matrix	Osteoid Cartilaginous Amorphous
MRI signal	Fat Fibrous/sclerotic Myxoid Hemorrhage Edema (bone marrow or soft tissues) Contrast enhancement

The radiological appearance of primary bone tumors can be summarized as follows:

- Osteosarcoma usually shows a characteristic destructive lesion in the metaphyseal area of long bones, mainly around the knee, proximal humerus or proximal femur. Mixed sclerotic or blastic matrix, and aggressive periosteal reaction (‘hair-on-end’ or ‘Codman’s triangle’) are common features in 75% of cases of conventional central osteosarcoma. Other histological subtypes can show quite different patterns, such as: (1) pure lytic lesions in telangiectatic osteosarcoma (frequently associated with fluid–fluid levels on MRI) or in fibroblastic subtypes [5]; (2) a solid bone mass growing from the superficial cortex as in parosteal osteosarcoma, a low grade variant which can be misdiagnosed as ossificans myositis or osteomas; (3) a lucent saucer lesion with an aggressive pattern on the surface of cortical bone, in periosteal osteosarcomas, mainly due to the presence of cartilage, and best characterized on MRI. In chondroblastic subtypes (conventional central or periosteal osteosarcomas), the differential diagnosis with chondrosarcoma can be challenging. Careful attention should be paid, when staging, to any evidence of satellite lesions or articular invasion, or any medullary invasion in juxtacortical variants, because of its surgical implications.
- Chondrosarcoma should be considered anytime there is a lytic, destructive lesion with amorphous, snowflake calcification, and mild or absent periosteal reaction in a patient over the age of 40. MRI helps to identify cartilage tissue with its typical lobular or ‘grape’ pattern on T2-SEFSWI and rim enhancement in CE-MRI. Differential diagnosis between enchondroma and low-grade chondrosarcoma needs a careful evaluation of clinical and radiological signs, but it is always challenging [6]. The proximal femur and pelvis are common places for this bone sarcoma. Presence of bimorphism (features suggesting a chondroid tumor adjacent to a markedly different area) should alert to a more aggressive high-grade or dedifferentiated chondrosarcoma [7]. CE-MRI may help to identify such areas, without the typical ring-enhancement pattern of low-grade cartilage, and it also allows a drive biopsy to be performed there.
- Chordoma affects the skull base, spine and sacrococcygeal area, the latter representing up to 50% of cases. A destructive lesion with soft tissue mass and intratumoral calcifications is the usual picture. Extension to adjacent vertebral bodies is common, as well as extension across the sacroiliac joint. MRI usually shows low to intermediate T1-SE WI signal, sometimes with areas of high signal intensity because of hemorrhage and high protein content; on T2-SE WI it shows a heterogeneous high signal intensity mass with a lobulated appearance and crisscrossing pattern [8]. CT shows a destructive lytic lesion with soft tissue mass, but MRI is preferred. Sometimes, CT can help to differentiate lytic small chordomas from the subtle sclerosing or normal appearance of benign notochordal lesions, previously detected on MRI. Differentiation from chondrosarcoma can be difficult, especially in the skull base.
- Giant cell tumor of bone (GCTB) typically exhibits pure osteolytic features with well-defined but non-sclerotic margins. GCTB is located eccentrically in the epiphyseal area of the bone, where it typically abuts the articular surface, and occurs almost exclusively in patients with closed epiphyses. Prominent trabeculation resulting in a multiloculated appearance is often seen. Regarding periosteal reaction, it is atypical with expanding radiolucent remodeling features, and sometimes, shows an aggressive appearance with cortical penetration and soft tissue extension. On MRI the tumor has from low to intermediate signal intensity on T1 and T2-weighted MRI, which helps to differentiate GCTB from other epiphyseal lesions or tumors, such as intraosseous ganglion or clear cell chondrosarcoma. A cystic appearance with fluid–fluid levels is present in 14% of cases, generally associated with secondary aneurysmal bone cyst [9].
- Other rare bone sarcomas:
  - Adamantinoma is a low grade tumor with epithelial and osteofibrous components that typically arises from the anterior tibial diaphysis (up to 90%), usually eccentrically or cortically based. It shows a geographic, bubbly lytic pattern with sclerotic margins in early disease, but it may appear more aggressive within a more advanced or recurrent disease.
  - Undifferentiated high-grade pleomorphic sarcoma (UPS) and spindle cell bone sarcomas (leiomyosarcoma and fibrosarcoma) produce neither osteoid or chondroid matrix nor reactive new bone. Therefore, they almost always appear as a lytic lesion with an aggressive permeative pattern, with spread into soft tissue boundaries. Dystrophic calcification may be seen in as many as 15% of cases. MRI shows nonspecific low signal on T1-SE WI and high signal on T2-SE WI, although it can sometimes show intermediate or low signal on T2-SE WI. Occasionally, an undifferentiated pleomorphic sarcoma is found to be a dedifferentiated chondrosarcoma or osteosarcoma after resection. Associations with pre-existing conditions (e.g., Paget’s disease or bone infarct) or previous irradiation have been reported.
  - Vascular bone sarcomas are a spectrum of low to high grade sarcomas. Hemangioendothelioma is a low or intermediate malignancy tumor, and angiosarcoma the high grade variant. They usually present as pure lytic lesions, sometimes multicentric, with variably aggressive tumor margins. MRI appearance is nonspecific, although vascular channels are occasionally seen and, unlike the benign hemangioma, they lack underlying fatty stroma. Interestingly, when they are multicentric, they tend to involve several bones of the same extremity [10].

### Biopsy

The goal of biopsy is to obtain diagnostic tissue while minimizing morbidity, limiting potential tumor spread, and avoiding interference with future treatments. Biopsy of a suspicious bone sarcoma must be carried out in a specialized center, once any other diagnostic/staging imaging procedures have been performed (IV, B). Changes secondary to tissue sampling can interfere with anatomic boundaries, tumor MRI signal and radionuclide uptake, making surgical planning or treatment response evaluation more challenging.

There are two types of biopsy procedure: incisional open biopsy (OB), and percutaneous imaging-guided core needle biopsy (CNB). Fine needle aspiration biopsy is not recommended as a first approach to suspected musculoskeletal sarcoma (IV, A) [11]. OB and CNB are equally valid when performed by an expert oncologist surgeon or a musculoskeletal radiologist, both integrated into a multidisciplinary sarcoma team (IV, A).

Some advantages make CNB the first choice technique. CNB can be performed on an out-patient basis under local anesthesia (with or without sedation). It has less post-procedural complications, it is cheaper, and it allows selection of the area of tumor with a more aggressive pattern by imaging (usually CT) (IV, A) [12]. Careful planning of the biopsy route with the oncologist surgeon is mandatory to ensure the biopsy tract is withdrawn en bloc during surgical treatment (tumor resection). There are some anatomic and compartmental boundaries that should be taken into account, as general rules, when CNB is performed to assure the best results in the planned surgery. At least three samples, whenever possible, are adequate to optimize diagnostic yield in CNB (IV, A) [13]. We recommend coaxial core biopsy needles, because they allow multiple samples in a single tumor approach, making CNB easier, faster and safer (V, A). When a bone tumor is associated with soft tissue mass, sampling the soft tissue mass alone can be easier, but it may add some diagnostic difficulties for the pathologist, difficulties which can usually be managed with an adequate radiologic–pathologic correlation.

OB allows larger samples of tissue, but it is more time consuming and requires general or regional anesthesia, with higher rates of tumor seeding and post-surgical complications compared to CNB (IV, A) [14]. Whenever possible it should be combined with intraoperative frozen sections to ensure that diagnostic material has been obtained. It is important to avoid any transversal incision, as well as to perform a careful surgical technique to prevent post-procedural hematomas, which should always be considered contaminated.

### Pathology

To make a diagnosis of a bone tumor it is essential to have the most relevant clinical and radiological information (patient’s age, size and location in the bone, radiological pattern and soft tissue extension) (IV, A). When the size or representativeness of the sample is not sufficient to reach a diagnosis, applying for a new biopsy is mandatory.

The samples must be sent fresh to the laboratory within half an hour of removal to preserve the integrity of nucleic acids. This procedure allows—before formalin fixation and decalcifying—the use of specific binding agents for histochemical or electron microscopy studies, microbiological culture or cytogenetic studies. Since decalcification procedures may negatively affect antigenicity and DNA quality, decalcification of bone biopsies with EDTA or formic acid agents (avoiding strong acids such as hydrochloric or nitric) is recommended (IV, A). Whenever possible, if it does not interfere with the diagnosis, it is recommended to freeze and preserve fragments of tumor tissue for molecular genetics studies and tumor biobank, as well as taking cytological imprints on slides for cytogenetic studies. The collection of frozen tissue material for further studies and research requires the completion of informed consent.

Microscopic examination of the hematoxylin-eosin stained slides, together with the integration of the clinical and radiological data, is still the basis of the diagnosis of bone tumors. The use of the World Health Organization (WHO) classification system is recommended, which incorporates morphological and genetic data and provides a uniform classification and nomenclature for the diagnosis of bone tumors [15]. In certain cases, the use of additional techniques such as immunohistochemistry (IHC) and/or molecular pathology is required for a definitive diagnosis. Genetic and molecular studies in the diagnosis of bone tumors are indicated in: unusual morphological variants, tumors with conventional morphology but with unusual clinicopathological presentation, as an indicator of response prediction to targeted therapies, and to meet criteria for inclusion in clinical trials.

The specific pathological characteristics of the main bone tumors are summarized below

- Conventional osteosarcoma is a high-grade tumor characterized by direct tumor osteoid and immature bone production by primitive sarcomatous cells. Depending on the proportion of osteoid, cartilage and/or fibroblastic tissue, it is subsequently subclassified into osteoblastic, chondroblastic and fibroblastic osteosarcoma. In the telangiectatic subtype, tumor osteoid and pleomorphic sarcoma cells are found in septum bordering blood-filled spaces. There are only two low-grade subtypes (low-grade intramedullary and parosteal osteosarcoma), both composed of relatively well developed trabeculae of woven bone in cellular spindle cell/fibroblastic background with mild cytologic atypia and low mitotic activity. Periosteal osteosarcoma shows predominantly chondroblastic differentiation (grade 2 or 3 chondrosarcoma) and is considered an intermediate-grade tumor.
- Conventional chondrosarcoma is a malignant cartilage tumor in which the tumor cells lie in lacunar spaces within the hyaline cartilage matrix, which may be partially calcified or myxoid, with or without foci of endochondral ossification. Isocitrate dehydrogenase (IDH)-1 and IDH-2 mutations can be found in primary and secondary chondrosarcomas. According to the degree of nuclear atypia, cellularity and mitotic activity, chondrosarcomas are classified into grades I, II or III. In dedifferentiated chondrosarcoma, a low-grade cartilaginous tumor coexists with a high-grade sarcoma with or without heterologous elements (undifferentiated pleomorphic sarcoma, osteosarcoma, rhabdomyosarcoma, etc.). Mesenchymal chondrosarcoma is a distinct tumor characterized by cartilaginous (low-grade) and non-cartilaginous elements, the latter being composed of solid and cellular areas of round or slightly spindled primitive mesenchymal cells resembling a small round cell tumor such as Ewing’s sarcoma.
- Chordoma, classified as an intermediate-grade tumor, has a lobulated architecture and is characterized by interconnecting cords of vacuolated or epithelioid cells in a prominent myxoid stroma, with a very low rate of mitotic activity. Chordoma may exhibit “cartilaginous-mimicking” matrix (chondroid chordoma) or, rarely, a high-grade spindle-cell or pleomorphic sarcoma (dedifferentiated chordoma). The latter is usually managed in the same way as soft tissue sarcomas. Immunohistochemically, chordomas coexpress S100 protein and epithelial markers (cytokeratins and epithelial membrane antigen—EMA). Brachyury is a more specific marker for chordoma, although it is lost in the dedifferentiated component.
- In giant cell tumor of bone the basic pattern is characterized by a stroma with oval or plump, spindle-shaped mononuclear cells with uniformly interspersed multinucleated giant cells. The nuclei of stromal and giant cells are similar. The mitotic rate of mononuclear stromal cells, which are considered to be the neoplastic component, can be quite high, but atypical mitoses are not present. Vascular invasion can be seen in 30% of cases. Despite these findings, there is no relationship between histologic criteria and clinical behavior. Histone-3-Family-Member-3A (H3F3A) mutation analysis appears to be a highly specific, although less sensitive, diagnostic tool for the distinction of GCTB from other giant cell-containing tumors. Interestingly, denosumab-treated GCTB shows marked giant cell depletion, although the impact on prognosis of this pathologic response is yet to be determined.

The pathology report of CNB should include histological type and grade, as well as the results of ancillary studies (IHC and/or molecular biology if performed) [1]. Unlike in soft tissue sarcomas, the grading system based on the degree of differentiation, mitotic index and percentage of necrosis has not proven to be useful in bone sarcomas and histological type itself determines the histologic grade except in chondrosarcoma, wherein the aforementioned histological parameters are still used to establish histologic grade (Table 2).

**Table 2:** Histological grading in bone sarcomas

Grade I
Parosteal osteosarcoma
Low-grade intramedullary osteosarcoma
Atypical cartilaginous tumor/ grade I chondrosarcoma
Clear cell chondrosarcoma
Grade II
Periosteal osteosarcoma
Grade II chondrosarcoma
Classic adamantinoma
Chordoma
Grade III
Osteosarcoma (conventional, telangiectatic, small cell, secondary, high-grade surface)
Undifferentiated high-grade pleomorphic sarcoma
Ewing sarcoma
Grade III chondrosarcoma
Dedifferentiated chondrosarcoma
Mesenchymal chondrosarcoma
Dedifferentiated chordoma
Malignancy in giant cell tumor of bone

The basic requirements for the pathology report of surgical specimens from bone tumors are: type of procedure, histological diagnosis (type and subtype), histological grade, measures of the surgical specimen (three dimensions in mm), presence of macroscopic tumor in the bone or soft tissue, size of the tumor in mm (three-dimensional), anatomic location in the bone, macroscopic appearance, local extent of the tumor, assessment of the status of resection margins, degree of local tumor spread and results of the ancillary techniques [1].

### Staging classifications and other initial assessments

Several staging systems for bone tumors are in use. The American Joint Committee on Cancer (AJCC) proposed a staging system based on TNM (Table 3) [16], although the most frequently used classification in bone tumors is the Enneking staging, developed in the 1980s and adapted by the Musculoskeletal Tumor Society (MSTS) (Table 4) [17]. Both systems consider histological grade. The AJCC staging also considers tumor size as a prognostic factor and it establishes a more accurate picture in different situations of disseminated disease. The Enneking staging, more surgically oriented, assesses the intra or extracompartmental location of the tumor, defining extracompartmental lesions as those in which the tumor infiltrates the periosteum and may even invade the adjacent soft tissues. Tumor burden and the presence of detectable metastases are the two main factors which are taken into consideration in the clinical staging of these diseases.

**Table 3:** AJCC Staging for bone sarcomas

Stage	Grade	Size	Metastases
IA	Low	< 8 cm	None
IB	Low	> 8 cm	None
IIA	High	< 8 cm	None
IIB	High	> 8 cm	None
III	Any grade	Any	Skip metastasis
IVA	Any grade	Any	Pulmonary metastases
IVB	Any grade	Any	Nonpulmonary metastases

**Table 4:** Enneking Staging for bone tumors

Stage	Grade	Site
IA	Low	Intra compartmental (T1)
IB	Low	Extra compartmental (T2)
IIA	High	Intra compartmental (T1)
IIB	High	Extra compartmental (T2)
III	Any grade	Any T, metastasis

Staging should be carried out to assess the extent of distant disease, including bone scintigraphy, and thoracic CT scan (IV, B). Whole-body MRI and positron emission tomography (PET)/CT or PET/MRI are under evaluation both for staging and treatment response evaluation. Additional appropriate imaging studies and biopsies can be taken from suspicious sites.

There are no specific laboratory tests for the diagnosis of bone sarcoma.

Chemotherapy treatment can result in renal, cardiac, and hearing impairment. Patients undergoing chemotherapy must have baseline renal function testing and assessment of cardiac function when anthracyclines are prescribed, as well as an audiogram in case of treatment with cisplatin.

Sperm or oocyte cryopreservation should be offered prior to starting chemotherapy.

## Treatment of specific histologies

### Osteosarcoma

Osteosarcoma is the most common primary malignant bone tumor in adolescents and young adults, although it has a second peak of incidence in older patients.

Most osteosarcomas are high-grade and require multimodal treatment, mainly chemotherapy and surgery. Low-grade osteosarcoma (including parosteal and low-grade central) does not require chemotherapy, but if the histological examination of a resected tumor shows high grade areas, chemotherapy should be recommended. The indication of neo/adjuvant chemotherapy in periosteal and craniofacial osteosarcoma is controversial, but it could be considered (IV, C). Adjuvant radiotherapy could also have a role in craniofacial location due to the difficulty of achieving wide margins (IV, C).

**Fig. 1:**
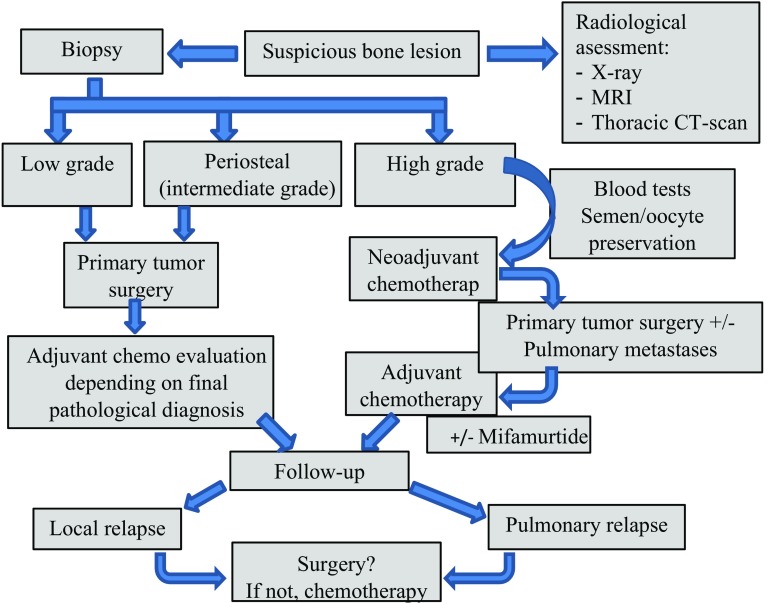
Diagnostic and therapeutic algorithm of osteosarcoma

In Fig. 1 an algorithm of the diagnostic and therapeutic process of osteosarcoma is provided.

There are several biomarkers that have been correlated to prognosis of high-grade osteosarcoma, such as *P*-glycoprotein, Her2, TP53 mutation and insulin-like growth factor-binding protein 3 (IGFBP-3), but none of them is prospectively validated and they are not routinely used in clinical practice [18–22].

The following recommendations are for the treatment of high-grade osteosarcoma. This tumor is considered a systemic disease, with high probabilities of subclinical metastases upon diagnosis of a localized tumor. Moreover, about 20% of patients are diagnosed with disseminated disease, for whom, despite its poor prognosis, cure is possible. Long-term overall survival (OS) decreases from approximately 60–70% in localized disease to 20–30% if the disease is disseminated [23].

### Therapeutic sequence

In the 1980s, the results of two randomized studies in osteosarcoma patients were published, showing a significant improvement in OS with adjuvant polychemotherapy (5 years OS increased from 20 to 60%) [24]. Since these results, adjuvant chemotherapy is absolutely recommended in high grade osteosarcoma (I, A).

Although it is usually recommended to begin chemotherapy before surgery, neoadjuvant therapy has not been shown to be superior to adjuvant treatment [25], and both options are considered valid (II, B). However, neoadjuvant treatment has a significant advantage because it allows in vivo testing of the sensitivity of the tumor to chemotherapy and could facilitate surgical resection (IV, B).

Neoadjuvant chemotherapy should be administered as soon as possible after histological diagnosis and staging. Intervals between chemotherapy and surgery, and surgery and resumption of chemotherapy should not be longer than 3–4 weeks. Longer delays might have an impact on disease-free survival (DFS) and OS (III, B) [26].

Among patients with metastatic disease at presentation, long-term survival rates were higher when metastases were removed versus when not (48 vs 5%) [27]. Metastases resection should be done after neoadjuvant chemotherapy, preferably after surgery on the primary tumor (III, A) [28].

### Initial chemotherapy scheme

There are different chemotherapy schemes described for osteosarcoma patients. The most active drugs are cisplatin, doxorubicin, high-dose methotrexate (HDMTX), ifosfamide and etoposide (I, A). Of these, cisplatin and doxorubicin are the most frequently included in the protocols. Most randomized trials of osteosarcoma have been developed in children and young adults (up to 35 years old), so few data exist in the middle-aged and older population.

In spite of the fact that phase III trials have not revealed the superiority of adding HDMTX to cisplatin–doxorubicin combinations, several studies including HDMTX have shown rates of DFS and OS at 5 years of over 60 and 70%, respectively [29]. These figures have never been achieved in studies of two-drug combinations alone, and for this reason HDMTX has been considered to form part of most of the protocols for children and young adults. The role of HDMTX in the rest of the adult population is still questioned due to the lack of data and its potential toxicity. When methotrexate is used in children and young adults the dose should be between 10 and 12 g/m^2^, but in patients over the age of 35 a reduction in that dose should be considered for reasons of safety. To avoid the high potential toxicity of HDMTX it is important to follow an exhaustive protocol (including adequate hydration, urine alkalization, folinic acid salvage and serial tests of methotrexate blood levels until negative results are achieved).

The MAP schedule (HDMTX, doxorubicin and cisplatin) is, therefore, considered the current standard treatment, at least in children and young adults, and is the option that has been used in the control arm of some of the most important randomized trials. The addition of ifosfamide to neoadjuvant treatment has not been shown to improve response rates, DFS or OS, and has been associated with increased hematological toxicity (I, A) [29, 30].

Based on all these considerations, the recommended initial neoadjuvant and/or adjuvant chemotherapy schedule, not only for localized but also for disseminated disease, would be the following for each age group:

- In adolescents and young adults up to 35-years-old: MAP schedule, considered in terms of efficacy to be superior to the cisplatin–doxorubicin combination (III, B). Recommended doses are listed in Table 5, although no full consensus exists as to the most appropriate administration regimen for each of the drugs.
- In middle-aged adults over 35-years-old: cisplatin–doxorubicin, although in some selected patients the MAP schedule could also be a valid alternative (IV, B). If HDMTX is used, extreme caution should be taken, and dose-reduction considered. Dosing of the cisplatin–doxorubicin combination is described in Table 6.
- In older adults decisions should be made on an individual basis, since the condition of most of these patients does not allow the use of cisplatin–doxorubicin, at least at the described doses.

**Table 5:** MAP chemotherapy schedule

Drug	Dose	Treatment weeks
Cisplatin	120 mg/m^2^ (60 mg/m^2^/day × 2 days) Alternatively: 100 mg/m^2^ × 1 day	Preop: weeks 1 and 6 Surgery: week 11 Postop: weeks 12 and 17
Doxorubicin	75 mg/m^2^ Original protocol: 37.5 mg/m^2^/day × 2 days in continuous infusion Alternatively: 25 mg/m^2^/day × 3 days in bolus form	Preop: weeks 1 and 6 Surgery: week 11 Postop: weeks 12, 17, 22 and 26
Methotrexate	12 g/m^2^ (up to a maximum of 20 g/cycle in 4 h × 1 day)	Preop: weeks 4, 5, 9 and 10 Surgery: week 11 Postop: weeks 15, 16, 20, 21, 24, 25, 28 and 29

This table indicates the dose and timing for each drug, as they were used in the control arm of randomised trials INT 0133 and Euramos-1. As explained in the text, no consensus has yet been reached about the best administration of this regimen, and certain variations may be considered valid

**Table 6:** Schedule for cisplatin–doxorubicin

Drug	Dose	Treatment weeks
Cisplatin	100 mg/m^2^ × 1 day Alternatively: 120 mg/m^2^ (60 mg/m^2^/day × 2 days)	Preop: weeks 1, 4 and 7 Surgery: week 9 Postop: weeks 11, 14 and 17
Doxorubicin	75 mg/m^2^ (25 mg/m^2^/day × 3 days in bolus form)	Preop: weeks 1, 4 and 7 Surgery: week 9 Postop: weeks 11, 14 and 17

### Evaluation of response to chemotherapy

Assessment of percentage of tumor necrosis in tumors treated with neoadjuvant chemotherapy is essential to check the effect of treatment and constitutes one of the most relevant prognostic factors (III, A). There is some evidence that dynamic imaging studies such as PET-TC or DCE-MRI correlate well with histological findings, which may give some idea of response before surgery (IV, B), but histology remains the mainstay technique to evaluate the response to treatment. Histological changes attributable to treatment in terms of necrosis, fibrosis, hyalinization, inflammation, hemorrhage and/or cystic change above 90% indicate a good prognosis and imply greater DFS [31].

### Surgery

#### Primary tumor resection

The local control of the tumor must be the primary therapeutic concern, and functional outcome is a secondary purpose. The location, extent of the tumor and functional priorities of the patient dictate the precise surgical techniques.

Local control is achieved by either limb-salvage surgery or amputation. Limb-salvage surgery is the preferred method if reasonable functional outcomes can be achieved after a negative surgical margin (IV, A). The limb-sparing surgery has two steps: Tumor resection and bone and soft tissue reconstructions. Amputation is considered in selected cases when curative surgery is possible and the resection to save the limb is unlikely to achieve a negative margin or a functionally viable extremity. Obstacles to limb salvage include poorly placed biopsy incisions, major vascular involvement, encasement of a major motor nerve, or pathological fracture of the involved bone. However, every indication should be individualized case by case [32, 33].

The incision must always be made along the main axis of the anatomical compartment containing the tumor and has to include the biopsy track en bloc, but there is still a debate about percutaneous tracts (IV, B) [34]. The margin is defined by the closest margin to any portion of the dissection. In this way, surgical resection can be classified as intralesional, marginal, wide, and radical. Only wide or radical resections are considered adequate for high-grade sarcoma.

There are three types of bone resection, depending on the anatomic site and the extent of the involved bone: intercalary (joint spared), intra-articular and extra-articular. When the tumor extends along the joint capsule or ligamentous structures or invades the joint, the entire joint needs to be resected, defined as extra-articular resection. Regarding soft tissue resections, the anatomical barriers (muscle fascia, adventitia, epineurium, and periosteum) have to be identified, and if these barriers are infiltrated the underlying structures should be resected en bloc with the tumor (IV, A).

Several reconstruction methods for limb-salvage surgery are possible and depend on the type of resection. The reconstruction can be achieved with structural allografts, metal endoprostheses, a combination or both (allografts–prostheses composite), and autografts (vascularized and nonvascularized). There are two types of structural allograft, intercalary and osteoarticular. The major advantage of allograft reconstruction is restoration of bone stock. Intercalary allograft reconstructions tend to perform better than osteoarticular grafts. Intercalary allograft is indicated after intercalary resections, and it is the key indication for reconstruction for this type of bone resection. After intra-articular resection, the reconstructions with allograft could be osteoarticular; however, the outcome is not as good as intercalary reconstructions, because the joint can be unstable. These reconstructions can be achieved by replacing the bone with an allograft, then substituting the joint with a total arthroplasty (composite) or modular oncology prostheses. There are some disadvantages to allografts when compared with metal endoprostheses; allografts must be fixed to the host bone and allowed to heal, and, consequently, they have to be protected from weight bearing for long periods of time. In addition, non-union and fractures may result in allograft failure.

The modular oncology prosthesis allows assembly of the necessary bone replacement to match the patient’s size and the amount of bone resected. The prosthesis can be cemented or uncemented. For younger patients uncemented is preferred, but which type of fixation is superior is debated. Complications can include loosening, wearing out of the parts, breakage and stiffness. Prosthesis is the preferred reconstruction method due to its reliably good outcome and the avoidance of delay in resumption of chemotherapy in high-grade sarcomas (IV, B).

#### Pathological fracture

A pathological fracture may lead to the tumor spreading into contiguous tissues, increase the risk of local recurrence and complicate the tumor margin. Limb-salvage surgery for selected patients with a pathological fracture, particularly one that is healing after chemotherapy, does not appear to increase the risk of local recurrence or death. If the patient receives neoadjuvant chemotherapy the pathological fracture needs to be immobilized (cast or external fixation). Open reduction and internal fixation are contraindicated. Therefore, in primary bone sarcomas pathological fracture is not an absolute contraindication for limb-sparing surgery, with the exception of chemoresistant tumors (IV, B) [35].

#### Pulmonary metastases

Surgical resection of pulmonary metastases should be considered as part of the treatment approach in cases of isolated lung metastases. OS at 5 years increases when full resection of all metastases is achieved, and reaches 72% in patients with less than three metastases resected from the lungs. Complete removal of all metastases must be attempted (III, B), as the disease is otherwise almost universally fatal, while more than a third of patients with a second surgical remission survive for more than 5 years [36].

The most frequent surgical technique is thoracotomy, although video thoracoscopy has achieved similar results in the presence of less than three nodes. Laser resection preserves a greater extent of healthy lung parenchyma and it has been shown to reduce postoperative complications and length of hospital stay when compared to traditional resection (III, B) [37]. If both lungs are involved, metastases from both sides must be excised whenever possible in the same surgical session, to continue or initiate postoperative systemic treatment as soon as possible. Cases of extended bilateral involvement may require surgery in two stages, with an interval of 3–4 weeks between each operation (IV, C).

### Postoperative systemic treatment

The standard adjuvant treatment is to continue with the same drugs that were used in the neoadjuvant setting. Results of studies evaluating chemotherapy-schedule modifications (especially with the addition of ifosfamide–etoposide) in poor responders to neoadjuvant therapy were controversial for many years. Recently, the results of a phase III trial (Euramos-1) evaluating this issue have been published and do not support the addition of ifosfamide and etoposide to postoperative chemotherapy in poor responders due to an increased toxicity without improving DFS [38]. Therefore, there is currently no evidence to support the modification of the chemotherapy scheme after surgery in poor responders (I, A).

The addition of mifamurtide to the MAP schedule after surgery improves OS at 6 years (78 vs 70%) without a statistically significant improvement in DFS, according to the results of a phase III trial including patients up to 30-years-old [29]. This drug was approved in Europe, but not in the US, to be added to adjuvant treatment of patients up to the age of 30 with localized disease (II, B).

### Radiation therapy

Though it is classically considered as a radioresistant tumor, new highly advanced technology, like intensity-modulated photon and charged-particle radiation techniques, are allowing the delivery of higher radiation doses to patients with bone sarcomas while simultaneously reducing the doses to critical normal tissue. These techniques may extend indications and are particularly promising for lesions in challenging axial sites where resections are often incomplete or associated with significant morbidity.

Radiotherapy can be an option as radical treatment of unresectable tumors, as adjuvant therapy when margins are positive, or as palliation of symptomatic metastases (IV, C) [39]. Some chemotherapy drugs (ifosfamide, cisplatin, HDMTX) seem to enhance the effectiveness of radiotherapy. For some patients, the combined approach of irradiation with chemotherapy may produce a long-term response.

Doses of postoperative radiotherapy vary between 45 and 50 Gy except in the case of patients with a positive margin, who should be treated with doses of 66–68 Gy. In the case of gross residual disease or inoperable osteosarcomas doses should be 74–76 Gy (70 Gy if concomitant chemotherapy) (IV, B).

### Relapse

About 30–40% of patients with localized disease will relapse (locally or as metastatic disease), and 80% of patients with metastatic disease will have a recurrence. The most frequent site for metastases is the lung; in the absence of additional visceral and bone metastases the disease could potentially be curable using surgery ± chemotherapy.

Local recurrence is treated with the same surgical approach as localized disease, although it has a worse prognosis (III, A) [36]. Patients with unresectable disease may benefit from radiotherapy. Chemotherapy after local relapse is not a standard treatment.

Treatment of recurrent metastatic osteosarcoma depends on the extension of metastases. If the disease is potentially resectable the initial approach should be surgical. Complete resection of metastases has been correlated with longer OS, not only in the first but also in subsequent recurrences (III, B) [36, 40].

The role of second-line chemotherapy is not well-defined. In one series the use of chemotherapy was correlated with longer OS in patients with unresectable disease or incomplete surgical resection (53 vs 12% of patients alive at 12 months), but the benefit was not clear after complete surgery (the chemotherapy only improved survival in the subgroup of patients with more than two pulmonary nodules) [41]. In another study, previously referred to, the benefit of chemotherapy was also superior in patients with incomplete resection [39]. Therefore, chemotherapy is recommended in patients with no complete resectable disease (III, C), and could be an option, though controversial, after complete removal of metastases (III, D).

The choice of chemotherapy scheme will depend on the treatment-free interval and the initial treatment received by the patient. If the patient received the MAP schedule previously, the recommended therapy includes a combination of ifosfamide and etoposide [42], with or without carboplatin at the doses described in Table 7 (III, C). If the patient was not given methotrexate for localized disease, HDMTX is another possible alternative.

**Table 7:** Schedules for IE (ifosfamide–etoposide) and ICE (IE + carboplatin)

Drug	Dose (mg/m^2^)	Days
Ifosfamide (w/mesna)	1800	1–5 every 21 days
Etoposide	100	1–5 every 21 days
± Carboplatin	400	1–2 every 21 days

**Fig. 2:**
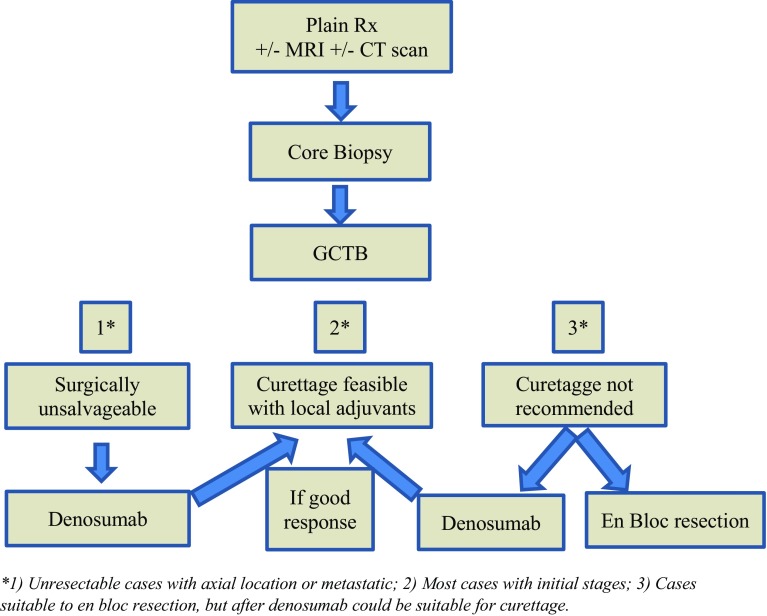
Algorithm for therapeutic decisions in GCTB

Phase II studies have shown that the use of the following drugs or drug combinations could have mild efficacy in poly-treated patients: docetaxel–gemcitabine [43], cyclophosphamide–topotecan [44], or sorafenib [45]. Hence, these treatments could be an option for selected patients with progressive disease after cisplatin, doxorubicin, HDMTX, ifosfamide and etoposide, knowing that they do not have the regulatory approval for this indication (III, C).

### Secondary osteosarcoma

Secondary osteosarcoma arises in previously irradiated sites or following a diagnosis of Paget’s disease. These osteosarcomas occur more frequently in regions other than the limbs and are more common in older patients. Five-year OS is 50% lower than in primary osteosarcoma, regardless of the tumor site or the previous use of radiation. The poorer prognosis of secondary osteosarcoma is related to the suboptimal surgical and chemotherapy treatment that usually affects these elderly patients. Several studies have shown that when secondary osteosarcomas are treated using a multimodal approach, with the doses and chemotherapy schedules of primary osteosarcoma, prognosis could be similar [46]. Therefore, treatment must be individualized on the basis of age, performance status, organ function and potential comorbidities. Whenever possible, treatment must consist of surgery and chemotherapy, as in primary osteosarcoma (III, C).

### Chondrosarcoma

Chondrosarcoma (CS) is the third most common primary malignancy of the bone after myeloma and osteosarcoma, and the most frequently occurring bone sarcoma in adulthood. The peak incidence is in the 5th–7th decades of life, although patients with secondary and mesenchymal CS are generally younger. The pelvis and the proximal femur (metaphysis) are the most common primary sites, followed by the proximal humerus, distal femur and ribs.

### Classification

Conventional CS accounts for about 85% of CS, but rarer subtypes include mesenchymal, (< 3%) and clear-cell chondrosarcoma (2%). Up to 10–15% of conventional CS can dedifferentiate into a very high-grade tumor with a dismal prognosis, the so-called dedifferentiated CS. Conventional CS can be divided into:

- Primary or central tumors, arising from previously normal bone.
- Secondary tumors that arise from previously existing benign cartilage lesions like enchondromas (secondary to central CS) or osteochondromas (secondary to peripheral CS), which are usually low grade tumors. Most chondrosarcomas are solitary, but they can occur as multiple lesions in patients with multiple osteochondromas and enchondromatosis. Patients with Ollier disease and Maffucci syndrome have an increased risk of secondary chondrosarcoma (40 and 53% respectively).

Fortunately, most chondrosarcomas arise as conventional, primary, low grade, locally aggressive, non-metastasizing tumors (grade I) rather than high grade (grades II–III). The 5-year OS for grade I CS is 83% while for grade II/III is 53%. Patients with dedifferentiated CS are more likely to develop metastases and have a dismal prognosis: 7–24% 5-year OS [47].

### Treatment

#### Surgery

The mainstay of treatment for CS is surgery and the approach is largely dependent on grade and anatomic location (see general principles of bone surgery described in osteosarcoma) (II, A). Nevertheless, low-grade intracompartmental CS of the extremities could be the exception and treated with extensive intralesional resection associated with local adjuvant treatment (high-speed burr, phenolization or cryotherapy, lavage with a high-pressure pulsatile system, and packing the defect with cement or bone graft) (III, A). An additional internal fixation could be indicated (mainly in the distal femur).

Generally, in conventional CS no adjuvant treatment is recommended, due to its low sensitivity to radio and chemotherapy (III, A). However, some retrospective reports suggest that mesenchymal CS is more chemo-sensitive, and may be considered for adjuvant or neoadjuvant therapy, mainly with Ewing-like regimens (IV, B) [48]. Additionally, in a retrospective review of 22 patients with dedifferentiated CS adjuvant treatment with CDDP and doxorubicin was associated with an improved OS (5-year survival 36%) [49], but this has not been confirmed in other studies (IV, C).

#### Radiation therapy

Regarding radiotherapy, it may be indicated in the case of an incomplete resection of a high-grade or mesenchymal CS (which are more radiosensitive than other subtypes) to maximize local control, and in situations where resection is not feasible or would cause high morbidity like spine and skull base (IV, B). Doses to achieve local control may exceed 60 Gy, so intensity modulated (IMRT), protons or brachytherapy could be an option with high local control rates (II, B) [50].

#### Inoperable or metastatic high-grade CS

These patients have a poor prognosis because of resistance to conventional treatments, such as radiotherapy and chemotherapy (III, B).

Regarding chemotherapy, a small phase II trial (25 patients) reported some activity (8% partial responses and 56% disease stabilization) of gemcitabine in combination with docetaxel (III, D) [51]. In addition, a retrospective study of 10 patients with recurrent conventional CS treated with cyclophosphamide and sirolimus reported 10% responses and 60% stabilizations with a progression-free survival of 13.4 months (IV, D) [52]. Chemosensitivity of dedifferentiated CS, which is often treated as a high-grade osteosarcoma, remains uncertain (IV, C) [49].

Palliative radiotherapy is also a reasonable option for local treatment of a primary or locally recurrent CS (IV, B) as long as sufficient doses are administered (40–70 Gy as monotherapy).

#### Relapse

In the case of recurrence, resection (local or pulmonary) should be the first option (IV, B). If it is unresectable, radiotherapy should be considered, especially in the case of skull base CS as previously mentioned (IV, B).

If local treatment is not considered feasible, systemic treatment within a clinical trial or with the previously described regimens should be discussed with the patient.

### Chordoma

Chordoma is a very rare tumor that arises from the embryonic remnants of the notochord and has a predilection for the axial skeleton, with the most common sites being the sacrum, skull base, and spine. Chordoma occurs in people of all ages with a median age of 60; skull base presentations can also affect the younger population. This is a low-grade tumor but locally aggressive, spreading throughout the neural structure and the axial skeleton. The metastases occur in 30% of cases and usually appear late in the natural history of disease, mostly after local recurrence.

### Treatment

Due to the rarity and long natural history of the disease, the quality of evidence available for chordoma is poor. The majority of published data are from case series and retrospective studies.

#### Surgery

Surgery is the mainstay of treatment and the goal is to achieve complete en-bloc resection without causing unacceptable harm. En bloc resection of the tumor in the first surgery provides the best chance for local control and long OS, surgical margins being the main prognostic factor (IV, B) [53, 54]. Tumor rupture must be avoided because it results in high risk of loco-regional recurrences.

Tumor location is the most important variable in defining the primary tumor treatment. For sacral chordoma, surgery should definitely be offered as the first choice when tumors arise from S3 or below, especially if preservation of S2 roots is possible, as it may result in some neurological recovery (associated with up to a 50% chance of normal bladder and bowel control) [53]. However, in tumors originating above S3, in the skull base or upper cervical tract surgical resection can be followed by important neurological sequelae, so other types of local treatment should be considered to preserve function.

#### Radiation therapy

If resection is not feasible, definitive radiation therapy alone should always be considered as a valid alternative (IV, B). Indications for definitive radiation therapy are: inoperable patients, neurological impairment not accepted by the patient or unresectable disease (IV, B).

Despite major advances in surgical interventions, total en bloc resection is only attainable in roughly 50% of sacral chordomas, with much lower rates for chordomas of the spine and skull base, meaning recurrence is common without en bloc resection. In these cases a maximally safe cytoreductive surgery plus high-dose radiation therapy should be considered [54].

The use of adjuvant radiation therapy in patients who have a complete resection is often advised because of the poor prognosis in those who relapse, however, its benefit is debated (V, C).

High doses of up to at least 74 Gy in conventional fractionation (1.8–2 Gy) are required to achieve adequate control of a chordoma [55]. Because of their proximity to critical organs radiotherapy must be highly conformal, meaning focused on the tumor while avoiding surrounding tissue. High-dose focused radiation with protons or photons [stereotactic radiosurgery (SRS) and IMRT] have allowed higher doses of radiotherapy to be delivered to the tumor while sparing surrounding structures. Hadron therapies, high-dose protons or carbon ions are the most recommended techniques for chordoma treatment because they provide improvement in dose conformation when compared to standard or 3D-conformal photon radiation therapy. Unlike photons, which lose their energy exponentially, after an initial energy-dependent build-up region, proton–matter interactions produce a superior dose distribution, by depositing the maximum dose at a specific depth determined by the proton beam energy (Bragg peak) (III, B) [56].

A systematic review of the literature analyzed seven retrospective studies that included a total of 416 patients with chordomas treated with protons or a combination of protons and photons. The radiation doses and schedules varied within and between series, but generally the total radiation dose was equivalent to 70 Gy or higher. At a median follow-up of 46 months, the 5-year local control and OS rates were 69 and 80%, respectively [57].

Data from a prospective study examining the outcome of proton therapy as either adjuvant or definitive treatment for nonmetastatic chordoma or CS reported local recurrence-free survival of 92%, with DFS of 87% [58]. Although promising, proton therapy is not widely available, in contrast with photon irradiation with image-guided intensity modulated radiotherapy (IG-IMRT). IG-IMRT for skull base chordoma can deliver high doses, equivalent to those typical of proton therapy [59], so it could be used in the case of unavailability of protons or carbon ions (III, B).

#### Recurrent and advanced disease

Despite best efforts at initial treatment, most chordomas will recur or progress. In the case of local relapse, the choice of treatment can include surgery and/or radiation therapy and/or systemic treatment, balancing morbidity and quality of life (V, C). Patients who have local recurrence have poor OS and they are unlikely to be cured by any local salvage treatment. Supportive care should be incorporated into the treatment from the beginning.

For patients with advanced disease or if surgery and/or radiation are not possible, systemic therapy can be used to slow tumor progression. Because chordoma is a slow-growing disease, classic chemotherapy is generally inactive. An exception may be high-grade dedifferentiated chordomas, a much more aggressive subtype, where schemes containing doxorubicin and ifosfamide can be used.

There are currently no approved drugs for the treatment of chordoma. A number of targeted agents have been evaluated to date in phase II trials with moderate efficacy, inhibiting specific molecules and their respective pathways known to be implicated in chordomas. These include PDGFR (imatinib) [60], EGFR (lapatinib, erlotinib) [61], mTOR (sirolimus), and VEGF (sunitinib, sorafenib) [62, 63] (III, C). Although all of them constitute active options, imatinib is the most frequently used drug and, therefore, we suggest using it first (IV, C). In a phase II trial of 56 patients with advanced chordoma treated with imatinib 70% of patients had stable disease, and the median progression-free survival was 9 months [60]. A watch-and-wait approach is also acceptable in the case of non-growing and asymptomatic tumors (IV, C).

### Giant cell tumor of bone

Giant cell tumor of bone (GCTB) is an uncommon, primary osteolytic tumor, typically affecting the epiphyses of long bones of skeletally mature patients of 20–40 years of age. Although the tumor is classified as benign, it tends to be locally aggressive and can metastasize, most often to the lungs. At presentation time around 20% of cases have pathologic fractures.

Recently, a driver mutation has associated GCTB with H3F3A in around 92% of cases [64]. Functionally, overexpression of Receptor Activator of Nuclear Factor Ligand (RANKL) by neoplastic mononuclear stromal cells is determinant for osteoclast-like cell recruitment [65]. The RANK pathway is the target of recently emerged new compound denosumab.

Malignant transformations, consisting of high grade sarcoma with no specific morphology, account for 8% in some large GCTB series. Most often they are secondary to transformations from previously irradiated GCTB. Less frequently, high-grade malignant tumors can arise from a conventional GCT at initial diagnosis. Rapid clinical and radiological changes in the context of recurrence, or lack of response to denosumab can signal a malignant tumor.

Due to the emergence of new therapies, the complexity of some cases, the clinical impact on the patient and the possibility of malignant transformation, cases with suspicion of GCTB should be presented to a multidisciplinary sarcoma board.

### Treatment

The algorithm for therapeutic decisions in GCTB is shown in Fig. 2.

#### Surgery

Surgery is the therapeutic mainstay of GCTB but it is crucial to discuss the cases before surgery to delineate the best therapeutic strategy. En bloc resections entail fewer recurrences (0–16%) but are related to higher functional impairment. Thus, extensive intralesional resection associated with local adjuvant treatment is the preferred surgical approach even when a higher recurrence rate is assumed (3–33%). Polymethyl methacrylate cementation (PMMA) alone after curettage seems to achieve the same local control as the additional application of phenol but there is not a general agreement (III, B) [66, 67]. In addition, chemical burns could be provoked in the surrounding tissues. Centers applying liquid nitrogen as adjuvant do not present better statistics in local control and the complication rates could be as high as 50% in some series, reporting postoperative fracture, skin necrosis, nerve palsy and infection. Nevertheless, no prospective comparative trials have addressed the efficacy of local adjuvant therapies in GCTB.

Curettage with PMMA can be repeated in the context of local recurrence with acceptable rates for subsequent recurrence (14–22%). However, it should be taken into account that lung metastases are more frequent in cases with multiple recurrences [67]. Importantly, recurrence is also correlated with location, thus in the distal radius the recurrence can be as high as 89%.

Axial GCTB constitutes a higher challenge due to involvement of nerve roots, spinal or pelvic instability and often because they are large tumors.

#### Systemic treatment

Bisphosphonates are stable analogue compounds of pyrophosphates conferring resistance to biological degradation. They have been used as adjuvant treatment after curettage [68] or as treatment for induction therapy [69], showing efficacy in the reduction of recurrences and in tumor control (III, B). Nevertheless, the studies were registered retrospectively with small series.

Denosumab is a fully human monoclonal antibody against RANKL, thereby inhibiting osteoclast-mediated bone destruction. Its activity in GCTB was well documented in the first proof-of-concept phase II trial with 37 GCTB patients. Histological assessment verified almost complete elimination of giant cells in all available cases. Radiologically, RECIST evaluation showed mostly stable disease with some patients achieving partial response. Likewise, early clinical benefit was registered including pain reduction, and improvements in function and bone repair [70]. The largest trial testing systemic treatment in GCTB tested denosumab in two different cohorts, the first enrolled 169 surgically unsalvageable cases and the second enrolled 100 cases where substantial morbidity was anticipated with surgery. The most relevant outcome consisted of 163 out of 169 patients from the first cohort being free of progression, 16 out of 26 patients underwent less aggressive surgery in the second cohort where 74% did not have surgery, with low toxicity [71]. As in the first trial, in this larger study an early clinical improvement was detected [72].

The scheme approved by the FDA and EMA for denosumab is 120 mg subcutaneous injection for 3 consecutive weeks and then monthly injections from week 5. Radiological evaluation after denosumab reveals bone repair and cortical restoration, which highlights the neoadjuvant value of denosumab especially in cases with cortical destruction. The impact on prognosis of the pathologic response to denosumab in GCTB is yet to be determined. Duration of neoadjuvant treatment is not established, but it depends on the bone restoration to facilitate curettage or resection, usually ranging between 4 and 8 months.

Therefore, indications for denosumab in the context of GCTB can be summarized as follows: as neoadjuvant to avoid or at least delay en bloc resection, facilitating extensive intralesional resection in cases of cortical destruction, and as treatment for disease control in axial or metastatic settings where surgery is not indicated or is disproportional (III, B).

#### Radiation therapy

Moderate dose (45–55 Gy) radiation therapy has been recommended in the past as a therapeutic approach in unresectable cases. However, most studies were retrospective with the inclusion of a small number of patients. The 5-year local control ranged from 62 to 90% [73]. Radiotherapy may be indicated only when complete excision or extensive intralesional resection is impossible due to medical or functional reasons or for aggressive, multiply recurrent GCTBs. Given that the typical unresectable GCTB is an axial lesion in a young person, proton therapy may represent a reasonable option (IV, B).

The induction of malignant transformation by radiation therapy in the context of GCTB is still a subject of debate, being 11% in some series. Nevertheless, with the emergence of denosumab, the role of radiation therapy should be redefined in those rare cases where denosumab may be ineffective.

### Other bone sarcomas

Soft tissue sarcomas (such as leiomyosarcoma, fibrosarcoma, UPS, etc.,) can metastasize to the bone. With a diagnosis of one of these rare histological subtypes in a bone biopsy, before considering a primary bone sarcoma it is advisable to rule out the possibility of a bone metastasis of a primary soft tissue sarcoma in another location.

### Undifferentiated high-grade pleomorphic sarcoma

UPS is typically high-grade with metastatic rates of at least 50%. Treatment usually involves neoadjuvant therapy followed by wide excision (IV, C). Its chemosensitivity and OS rate are similar to those of osteosarcoma. Occasionally, an undifferentiated pleomorphic sarcoma is found to be a dedifferentiated chondrosarcoma or osteosarcoma after resection.

### Spindle cell bone sarcoma

These represent between 2 and 5% of primary bone malignancies, and include leiomyosarcoma and fibrosarcoma. They arise in a similar age group to CS, with a skeletal distribution similar to osteosarcoma. They are more likely to be pure lytic lesions, which may account for the high incidence of pathological fracture. Associations with pre-existing conditions (e.g., Paget’s disease or bone infarct) or previous irradiation have been reported [74].

These patients should be treated in the same way as those with an osteosarcoma, with pre-operative chemotherapy and wide surgical resection (IV, C). Most of the classic prognostic factors that have been identified for osteosarcoma seem to apply to spindle cell sarcomas [74].

### Fibrosarcoma

Surgery is the treatment of choice. With a high probability of metastases (> 70%) after surgical treatment, perioperative adjuvant treatment modalities should be considered for high-grade tumors (IV, C) [75].

Bone sclerosing epithelioid fibrosarcoma is a very rare malignant neoplasm, related to low grade fibromyxoid sarcoma. Demonstration of Fused in Sarcoma (FUS) or Ewing Sarcoma RNA Binding Protein 1 (EWSR1) rearrangements can help support the diagnosis, as does the combination of Mucin 4 (MUC4) positivity and Special AT-rich sequence-binding protein 2 (SATB2) negativity by immunohistochemistry. Treatment should be surgical. Chemotherapy and radiation have been used in case reports, but their efficacy has not been established [76].

### Leiomyosarcoma

Primary leiomyosarcomas are usually treated like osteosarcoma. They might have a slightly better prognosis than patients with osteosarcoma. A recent Japanese series reported a 5-year OS and DFS of 78.3 and 44.9%, respectively. Treatment should include en bloc removal with clear margins. Cisplatin-based neoadjuvant regimens have suboptimal efficacy. The optimal chemotherapy regimen remains to be established (IV, C) [77].

### Vascular tumors

Vascular tumors of the bone include a spectrum of lesions ranging from benign hemangiomas to malignant angiosarcomas, with subcategories of hemangioendothelioma representing tumors of intermediate biological behavior [78].

Primary angiosarcoma of the bone is a rare, aggressive, high-grade malignant neoplasm with endothelial differentiation and a very high mortality; 1- and 5-year survival rates are approximately 55 and 33%, respectively. Approximately 30% are multifocal. Most cases present in adults over 30 years of age. Occasional cases have been reported subsequent to radiation therapy [78]. Optimal therapy beyond surgery has not been established (IV, C).

### Adamantinoma

Adamantinoma is a low-grade malignancy with metastatic potential in 15–30% of patients, and it can be fatal. Local recurrences may occur in up to one-third of patients, and can be of very late onset.

Chemotherapy and radiation have not been effective in this tumor. Surgical management is necessary, with the goal of attaining clear margins (IV, C). Historically, amputation was the treatment of choice. In a recent series en bloc resection with wide margins, followed by appropriate reconstruction have been associated with a low risk of local recurrence, a final limb preservation rate of 84% and 10-year survival rate of 87%. Metastases are managed with surgical resection.

## Follow-up

To detect local relapse or metastatic disease, after initial treatment, follow-up is designed based on the assumption that early diagnosis can lead to early effective treatment, and therefore, better survival.

Follow-up of patients with bone tumors after finishing initial treatment should include:

- Physical examination, functional assessment, and possible complications of any reconstruction (IV, D) [79].
- Radiological examination of primary tumor site: X-ray and/or MRI are the most used tools, although the role of regular cross sectional imaging remains uncertain. PET scans are now under investigation, but can help in specific cases, when other tests give confusing information (III, C).
- Chest X-ray/CT scans to detect early lung metastases. Based on prospective data CT scans are superior to plain chest X-ray in detecting early lung metastases, but without an improvement in OS (I, B) [80].
- Bone scintigraphy: although in osteosarcoma even lung metastases can be detected by this method, it is only used when bone metastases are suspected (III, C).

Based on the natural history of these diseases, follow-up time interval frequency after completion of initial treatment could be:

- Every 3 months for the first 2 years.
- Every 4–6 months from 2nd to 5th year.
- Every 12 months from 5th to 10th year.

The scarce prospective data did not demonstrate non-inferiority of 6-month against 3-month intervals in the first few years [80]. In the case of low-grade bone sarcoma (including GCTB, chordoma and chondrosarcoma) follow-up visits can be less frequent, but no specific recommendation can be given. Although late recurrences beyond 10 years are reported, surveillance more than this time is not certain to be useful.

Long-term toxicity of chemotherapy and radiotherapy must also be carefully monitored in follow-up [81]. Special attention must be paid to secondary neoplasms that can be increased for at least 25 years after diagnosis [82].
